# Comminuted nasogastric tube fracture: a rare complication

**DOI:** 10.1259/bjrcr.20210049

**Published:** 2021-05-12

**Authors:** Hiba Eldigair, Ruhaid Khurram, Jose Bennell, Naheed Mir

**Affiliations:** 1 Royal Free London NHS Foundation Trust, London, UK

## Abstract

A nasogastric tube is commonly used as a method of enteral feeding or gastric decompression in clinical practice and its insertion is occasionally associated with local complications. In this case report, we present an extremely rare complication of a comminuted nasogastric tube fracture in a 54-year-old male patient receiving enteral feeding in hospital secondary to a diagnosis of haemophagocytic lymphohistiocytosis.

## Introduction

Nasogastric tubes are frequently placed as a temporary support measure for administration of nutrition and/or medications in patients who are unable to tolerate oral intake but have a preserved functional gastrointestinal tract. Long-term nutritional support solutions include gastrostomy or jejunostomy feeding tubes and are reserved for patients who do not recover their swallowing function.^1^ Another common indication is to decompress the stomach in the setting of small or large bowel obstruction, in which a wide-bore Ryles tube is preferentially utilised. Common complications of NG tube insertion include local irritation, sinusitis, sore throat and epistaxis. Respiratory placement, pulmonary injury, aspiration, luminal perforation and intracranial placement are more serious noted adverse events.^2^

The fracture or transection of a NG tube is rare with a scarce number of cases reported in the literature.^3–7^ We describe a case of a 54-year-old male patient who was incidentally found to have a comminuted fracture of his NG tube following a long hospital admission for an aggressive systemic inflammatory disorder called haemophagocytic lymphohistiocytosis (HLH).

## Case presentation

A 54-year-old male presented to hospital with a 6-week history of weight loss (9 kg, was 61 kg at time of report), lethargy and dysphagia. He reported 1 week of loose bloody stools (three times/day) and a single episode of non-bloody vomiting 1 day prior to admission. His past medical history included ischaemic heart disease having suffered a non-ST elevation myocardial infarction requiring angioplasty and stenting in 2009, anxiety and depression. He was a smoker (approximately 1 pack of 20 cigarettes per day for 25 years), worked as a decorator and lived with his wife and two sons.

Blood tests revealed a pancytopaenia with an elevated inflammatory response. Viral serology results for Hepatitis B/C, Human Immunodeficiency Virus (HIV) and Epstein-Barr Virus (EBV) were negative. Gastroscopy revealed a 5 mm duodenal ulcer, multiple gastric erosions, two small Mallory-Weiss tears and stigmata of recent haemorrhage. No active bleeding point was seen. High-dose proton pump inhibitor therapy was initiated.

A CT scan of the thorax demonstrated multiple bilateral lung nodules and an enlarged right hilar lymph node. He underwent a fludeoxyglucose (FDG)-positron emission tomography (PET) scan which showed progression in pulmonary nodules, avid intrathoracic and subdiaphragmatic lymph nodes and increased FDG activity in the spleen, bone marrow and reactive nodes. These findings were suggestive of a systemic inflammatory response or a lymphoproliferative disorder.

A subsequent bone marrow biopsy was performed revealing mild to moderate haemophagocytosis and no evidence of a malignancy. A diagnosis of HLH was made and immunosuppressive treatment commenced with a regime of intravenous methylprednisolone and immunomodulating therapy.

As a consequence of the systemic inflammatory disorder, his oral intake had significantly reduced and an 8 French (Fr) NG tube was inserted to provide nutritional supplementation and medication. The appropriate positioning of the tube was radiologically confirmed and enteral feeding proceeded uneventfully. Intravenous proton pump inhibitor (PPI) was being used which increased gastric pH. This resulted in more than the usual number of chest radiographs to confirm the NG position (4 times in 2 weeks). All of these showed an intact NG tube.

**Figure 1.**
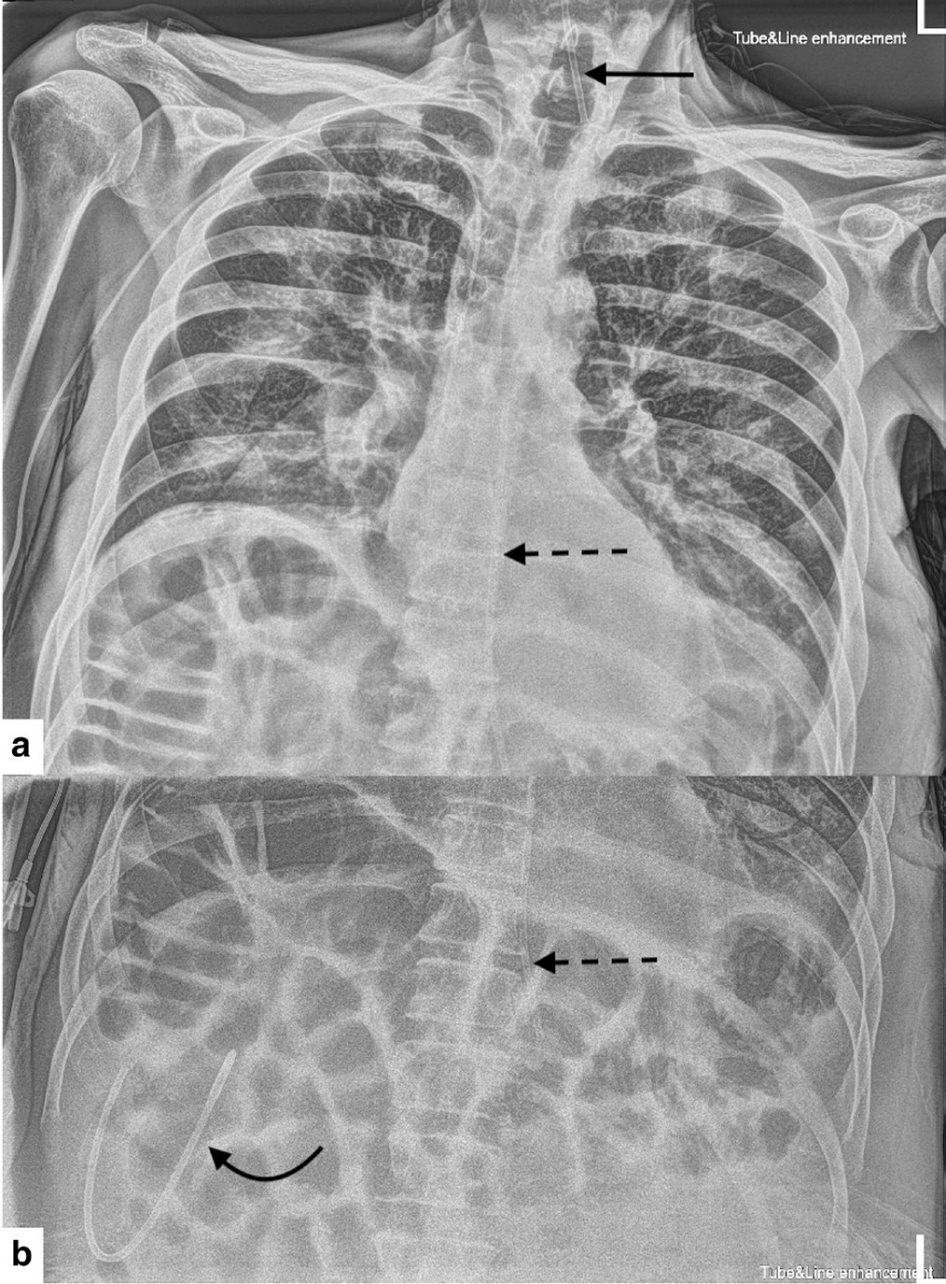
**A**) Anteroposterior chest radiograph with tube and line enhancement windowing demonstrating the distal tip of the NG tube proximal segment projecting medial to the left clavicle (straight arrow). The middle segment with its proximal tip projecting just below the left main bronchus (dashed arrow). (**B**) Subdiaphragmatic view. The distal tip of the middle segment is situated at the GOJ (dashed arrow) and the distal end of the NG tube is demonstrated below the right hemidiaphragm (curved arrow). GOJ, gastro-oesophageal junction; NG, nasogastric.

2 weeks following insertion of the NG tube, the nursing staff started the feed as usual in the evening and documented “NG *in situ* and NG feed running fine”. Overnight, the nurse documented that the patient had reported discomfort on feeding, so it was temporarily halted. Nursing staff were unable to obtain an aspirate despite multiple attempts at aspirating the tube, hence a repeat chest radiograph was performed to determine tube position. This revealed that the NG tube had split into three segments: the distal tip of the proximal segment was projected medial to the left clavicle, a middle segment with its distal tip at the gastro-oesophageal junction and the distal end of the NG tube separated and situated below the right hemidiaphragm (Figure 1). This was in keeping with a comminuted fracture of the NG tube.

**Figure 2.**
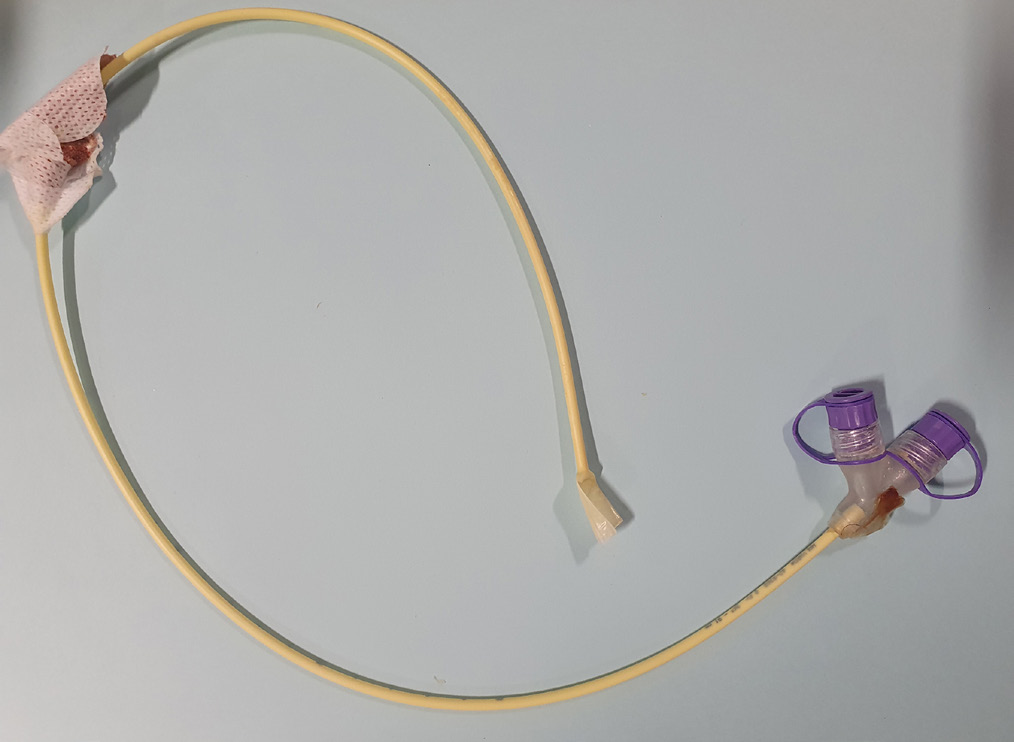
An image of the manually retrieved proximal NG tube fracture segment. NG, nasogastric.

**Figure 3.**
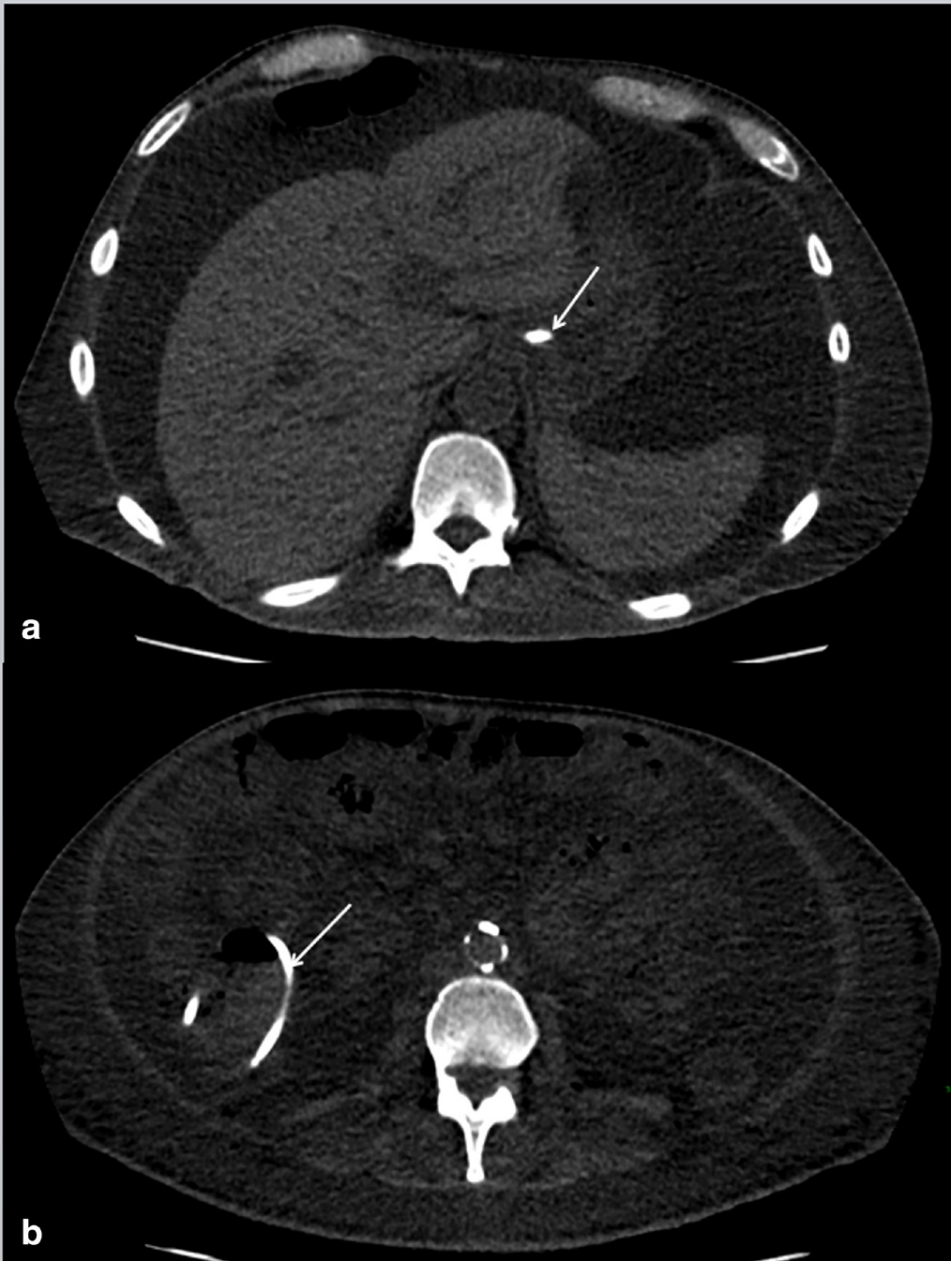
CT thorax abdomen and pelvis in axial slices and soft tissue windowing. Interval removal of the proximal segment of the fractured NG tube. (**A**) Middle segment of the fractured NG tube is demonstrated within the distal oesophagus and proximal stomach. (**B**) The distal segment is within the ascending colon. Diffuse ascites is also demonstrated. No evidence of perforation or obstruction. NG, nasogastric.

A CT thorax, abdomen and pelvis was performed to evaluate the precise location of these fragments. The proximal segment had been manually removed in the interim (Figure 2). The middle segment was within the distal oesophagus and stomach, and the distal segment within the ascending colon (Figure 3). There were no reported complications on CT secondary to the fractured segments, *e.g*. perforation or obstruction. There were also no features of aspiration pneumonia within the chest. The patient remained clinically asymptomatic from this NG tube complication.

**Figure 4.**
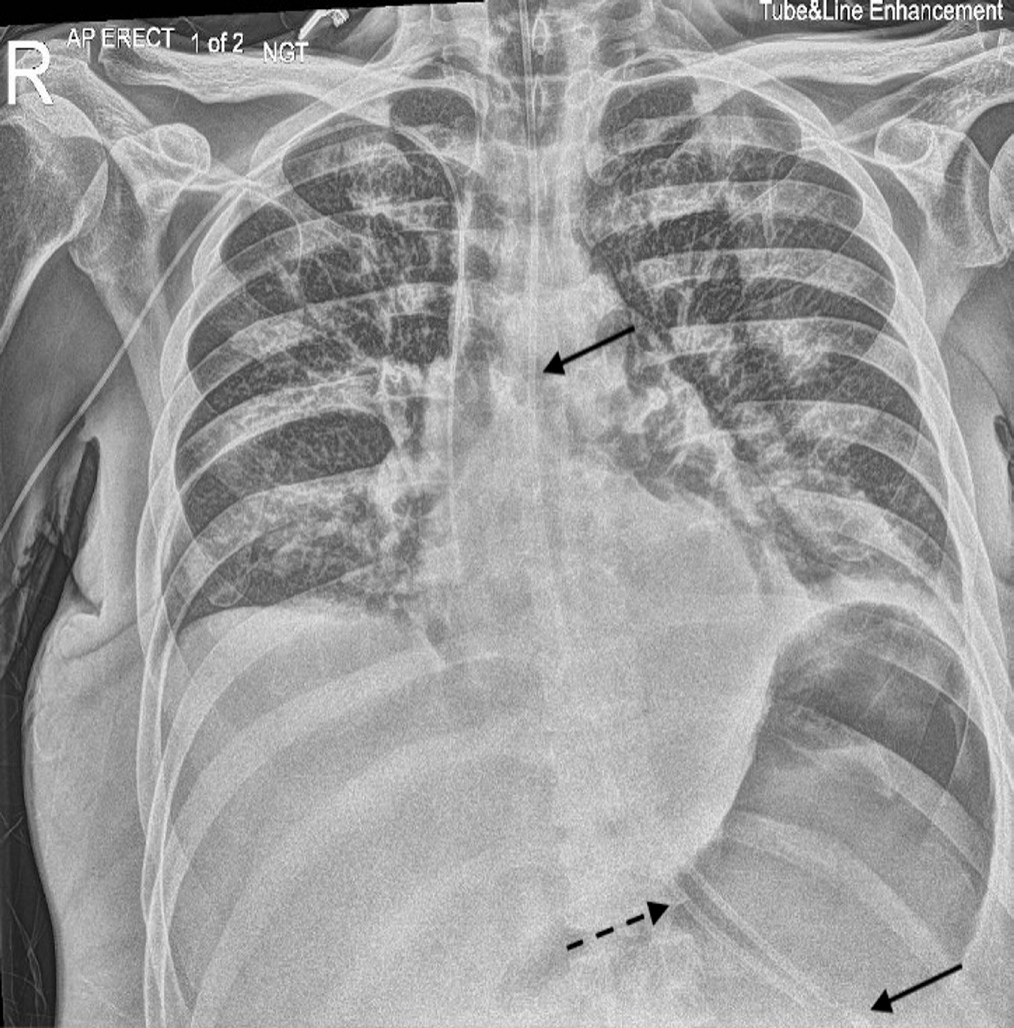
Anteroposterior chest radiograph with tube and line enhancement windowing the following day demonstrating a new correctly positioned NG tube with its tip below the left hemidiaphragm in the region of the stomach (straight arrow). Adjacent to this is the fractured middle segment of the previous NG tube situated near the GOJ with its distal tip visualised in the stomach (dashed arrow). GOJ, gastro-oesophageal junction; NG, nasogastric.

**Figure 5.**
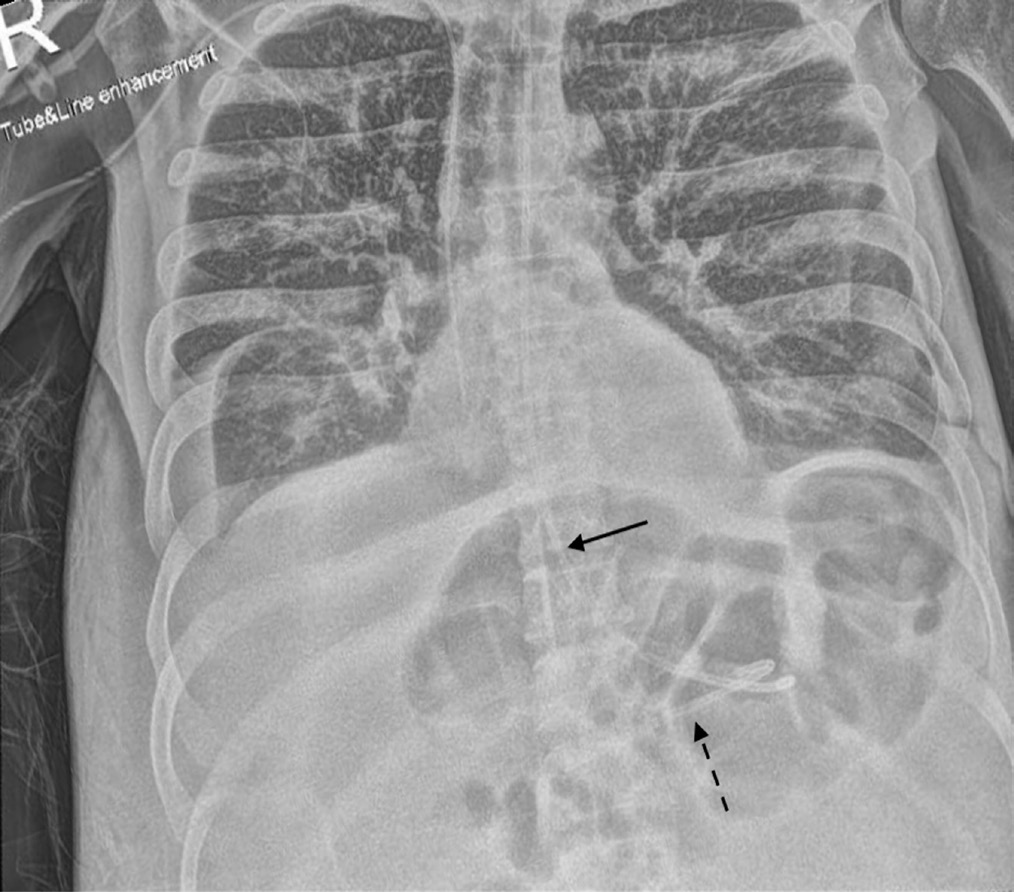
Anteroposterior chest radiograph with tube and line enhancement windowing two days later demonstrating the previously fractured middle segment of the NG tube in the upper abdomen, within the region of the stomach (dashed arrow) and the new correctly positioned NG tube (straight arrow). NG, nasogastric.

The gastroenterology and nutrition team were consulted, who advised conservative management as the remaining segments were likely to spontaneously pass through the gastrointestinal tract given their position. A new 8 Fr NG tube was inserted without any complication the following day. Subsequently, a chest radiograph demonstrated the fractured middle segment of the NG tube in the gastro-oesophageal junction near the new correctly positioned NG tube (Figure 4). Further imaging 2 days later revealed that the fractured middle segment had migrated slightly caudally into the upper abdomen, presumably within the stomach (Figure 5). Therefore, the fractured middle segment did not cause an obstacle to the placement of the new NG tube.

The patient’s condition continued to deteriorate as a result of the systemic inflammatory response from HLH. He developed profound hypoalbuminaemia despite enteral feeding leading to the development of anasarca. He unfortunately passed away during the hospital admission secondary to the effects of pulmonary oedema.

## Discussion

NG tubes are traditionally inserted at the patient’s bedside and prior to the administration of medications or nutrition, intragastric placement of the NG tube must be confirmed. The first-line method to confirm correct positioning of the NG tube is pH testing of an aspirate. The National Patient Safety Agency of the United Kingdom states that a pH of 5.5 or less confirms safe positioning. If sampling of a NG tube aspirate is unsuccessful or the pH test is inconclusive, correct positioning of the NG tube is confirmed via a chest radiograph which is also the gold standard evaluation method.^8^

A NG tube must fulfil certain criteria on a chest radiograph for it to be classed as appropriately sited. It should bisect the carina or the left main bronchus, have a midline intrathoracic course and its tip should be clearly visualised below the left hemidiaphragm. Current guidelines also recommend radiologists to explicitly document the position of the nasogastric tube tip and whether or not it is safe to proceed with feeding.^8,9^

Local discomfort, sinusitis, sore throat and epistaxis constitute some of the common complications that may arise following nasogastric tube insertion. Misplacement of the nasogastric tube, especially within the bronchial tree, is a serious complication which can subsequently result in aspiration if not recognised in a timely manner. Introducing feed, flush or medication into the respiratory tract or pleura through a misplaced nasogastric or orogastric tube was confirmed by the Department of Health in England as a ‘never event’ in 2011. It is advised that misplacement incidents must be reported locally as well as nationally to the NRLS (a central database of patient safety incident reports).^8,9^

Fracturing of the nasogastric tube is a very rare complication. To our knowledge, there have only been four previous cases reported in the literature.^3–7^ The precise mechanism for how and why this occurs is not fully understood; however, there are a few important risk factors which may explain this phenomenon. Although NG tubes are made with durable and malleable plastic, inherent manufacturing defects may predispose to fracturing especially in a comminuted manner. Furthermore, NG tube occlusions often cause a hindrance to timely feeding and medicinal administration. The nursing staff responsible for our patient reported this as a recurrent problem and the NG tube required repeated unblocking. This may have been secondary to clumping of medication during administration since the patient was on a complicated treatment regimen. We can therefore postulate that rising intraluminal pressures as a result of flushing a frequently blocked NG tube, may have contributed to fracturing secondary to possible weakening of the NG tube wall. Other risk factors include traumatic initial insertion or flushing a knotted NG tube,^7,10,11^ both of which did not directly apply in our case.

Although guidelines recommend urgent endoscopic retrieval for foreign objects >6 cm in size situated proximal to the duodenum,^12^ an individualised approach to management should be considered especially in the context of patients who are clinically unfit for endoscopy. Both conservative and endoscopic treatments have been described in the literature.^4,5^ In our case, a multidisciplinary decision was made to manually retrieve the proximal fragment to prevent local discomfort to the patient however, since the patient was too unwell for invasive intervention, the middle and distal fragments were left to pass spontaneously.

## Learning points

- The fracture or transection of a NG tube is a rare associated complication of insertion.
- It is important for clinicians and radiologists to be aware of this complication for appropriate identification and to prevent unsafe enteral feeding.
